# CIP2A mediates fibronectin-induced bladder cancer cell proliferation by stabilizing β-catenin

**DOI:** 10.1186/s13046-017-0539-8

**Published:** 2017-05-18

**Authors:** Fengbin Gao, Tianyuan Xu, Xianjin Wang, Shan Zhong, Shanwen Chen, Minguang Zhang, Xiaohua Zhang, Yifan Shen, Xiaojing Wang, Chen Xu, Zhoujun Shen

**Affiliations:** 1 Department of Urology, Ruijin Hospital, School of Medicine, Shanghai Jiaotong University, No.197 Ruijin 2nd Road, 200025 Shanghai, China; 2 Department of Urology, Huashan Hospital, Fudan University, No.12 Middle Urumqi Road, 200040 Shanghai, China; 3 Shanghai Key Laboratory of Reproductive Medicine, School of Medicine, Shanghai Jiaotong University, No.227 South Chongqing Road, 200025 Shanghai, China

**Keywords:** Bladder cancer, Fibronectin, CIP2A, β-catenin, Proliferation

## Abstract

**Background:**

Fibronectin (FN) is associated with tumorigenesis and progression in bladder cancer, however, the underlying mechanisms causing this remain largely unknown. Furthermore, cancerous inhibitor of protein phosphatase 2A (CIP2A) has been shown to play important regulatory roles in cancer proliferation. Here, we investigated whether FN regulates CIP2A expression to promote bladder cancer cell proliferation.

**Methods:**

The correlations of stromal FN with CIP2A and proliferating cell nuclear antigen (PCNA) expression were analyzed in a cohort bladder cancer patients. The roles of FN and CIP2A in regulating bladder cancer cell proliferation were evaluated in cell and animal models. Cycloheximide treatment was used to determine the effects of CIP2A on β-catenin stabilization. The CIP2A-β-catenin interaction was confirmed by immunofluorescence staining and co-immunoprcipitation.

**Results:**

In this study, we found that stromal FN expression correlated positively with the levels of CIP2A and PCNA in bladder cancer tissues. Meanwhile, in human bladder cancer cell lines (T24 and J82), exogenous FN significantly promoted cell proliferation, however, CIP2A depletion inhibited this process. Furthermore, the interaction between CIP2A and β-catenin enhanced the stabilization of β-catenin, which was involved in FN-induced cell proliferation. In vivo, CIP2A depletion repressed FN-accelerated subcutaneous xenograft growth rates.

**Conclusions:**

These data reveal that CIP2A is a crucial mediator of FN-induced bladder cancer cell proliferation via enhancing the stabilization of β-catenin. Promisingly, FN and CIP2A could serve as potential therapeutic targets for bladder cancer treatment.

**Electronic supplementary material:**

The online version of this article (doi:10.1186/s13046-017-0539-8) contains supplementary material, which is available to authorized users.

## Background

Due to the high morbidity and mortality, bladder cancer is the most common urologic malignant tumor, which is divided into two types, non-muscle-invasive bladder cancer (NMIBC) and muscle-invasive bladder cancer (MIBC) [1]. However, NMIBC is characterized with high recurrence rate and a 20% chance of progression [2], meanwhile, a poor 5-year survival rate of less than 60% is observed in the MIBC patients who have undergone radical cystectomy and systematic chemotherapy [3]. Therefore, biomarkers for early detection and key elements for targeted therapy are urgently demanded for bladder cancer treatment.

Extracellular matrix (ECM) microenvironment possesses a vital role in regulating cancer cell behaviors by causing a disorder of cancer-related gene expression [4]. Fibronectin (FN), an essential component of ECM, has been found highly expressed in several types of cancer, indicating a potential role of FN in tumorigenesis and progression [5, 6]. Actually, evidences have shown that the levels of FN are implicated in bladder cancer stages and grades [7, 8]. A systematic review with meta-analysis conducted by Yang et al. has revealed that urine FN has a pooled sensitivity of 81% and a pooled specificity of 80% in diagnosing bladder cancer [9]. Moreover, an in vitro study has demonstrated that FN protects bladder cancer cells from mitomycin C-induced cell death through inhibition cell cycle arrest [10], which implies the latent relationship between FN and cell proliferation in bladder cancer.

Unconstrained cell growth plays a key role in the initiation and progression of malignant tumors. Aberrations of various crucial signaling cascades associated with sustained proliferation is hallmarks of many malignancies [11]. One such signaling cascade is the Wnt/β-catenin pathway, which has been confirmed to exert a profound effect on bladder cancer cell proliferation [12]. In the Wnt pathway, β-catenin and its downstream target proteins, CyclinD1 and c-myc, are important regulators in cell proliferative activity [13].

Cancerous inhibitor of protein phosphatase 2A (CIP2A), a human oncoprotein dysregulated in several cancer types, is supported to have prominent effects on cancer cell proliferation [14]. CIP2A depletion reduces both in vitro malignant cellular growth and in vivo xenografted tumor formation [15, 16]. In addition, CIP2A has been proved to regulate cell-cycle progression [17]. Although the relationship between the overexpression of CIP2A and aberrant cell proliferation in bladder cancer has been confirmed previously [18], the cause of CIP2A overexpression and the mechanisms though which CIP2A exerts its proliferative properties in bladder cancer are still unclear.

In this context, the present study is designed to explore the significance and therapeutic potential of FN-CIP2A-β-catenin signaling pathway in bladder cancer. Here we demonstrate the positive correlations of stromal FN with CIP2A and PCNA expression in a cohort human bladder cancer tissues. Moreover, we validate that FN promotes bladder cancer cell proliferation by increasing CIP2A expression in cell and animal models. Furthermore, our results strongly indicate that CIP2A-β-catenin interaction regulates the stability of β-catenin which is involved in the process of FN-induced bladder cancer cell proliferation.

## Methods

### Patients and tissue samples

Human bladder cancer samples were obtained from 68 patients who underwent transurethral resection of bladder tumor (TURBT), partial or radical cystectomy in Ruijin Hospital (Shanghai, China) and Huashan Hospital (Shanghai, China), between September 2015 and September 2016. According to the collected pathological information, a total of 39 patients were NMIBC, and 29 patients suffered from MIBC. All procedures of this study were approved by institutional ethics committees of the above two hospitals. In accordance with the Declaration of Helsinki, the research was carried out, and written informed consent was obtained from each patient.

### Cell culture and chemicals

Human bladder cancer cell lines (T24 and J82), obtained from Type Culture Collection of the Chinese Academy of Science (Shanghai, China), were cultured in DMEM (HyClone, USA) containing 10% fetal bovine serum (Gibco, USA), 100 units of penicillin/mL and 100 μg of streptomycin/mL. Cells were cultured at 37 °C with 5% CO2. FN from human plasma (Sigma, Japan) was dissolved in culture media with different concentrations (0, 5, 10 and 20 μg/mL) for cell incubation. To measure endogenous β-catenin stability, cells were incubated with 100 μg/mL cycloheximide (CHX) (Sigma, Japan) for the indicated times.

### RNA extraction and quantitative real-time PCR (qRT-PCR)

Total RNA was extracted by using Trizol reagent (Invitrogen, USA), and PrimeScript^TM^ RT Master Mix (TaKaRa, Japan) were used for the reverse-transcription reactions. The following primers were used for amplification: CIP2A, forward primer (5’-CACAAAT-CACCTCGACCCCT-3’) and reverse primer (5’-CAAAAGCTGAGTGGCGTTCG-3’), β-catenin, forward primer (5’-GCGCCATTTTAAGCCTCTCG-3’) and reverse primer (5’-GGCCATGTCCAACTCCATCA-3’), GAPDH, forward primer (5’- ACCA-CAGTCCATGCCATCAC -3’) and reverse primer (5’- CCACCACCCTGTTGCTG -3’) and β-Actin, forward primer (5’-GTGGGGCGCCCCCAGGCACCA-3’) and reverse primer (5’-CTCCTTAATGTCA-CGCACGAT-3’). QRT-PCR was carried out using SYBR Premix Ex Taq II Kit (TaKaRa, Japan) on an Applied Biosystems 7500 Real-time PCR system (Applied Biosystems, USA). GAPDH and β-Actin were used as internal control. The results were expressed as log_10_ (2^-ΔΔCt^).

### Western blotting analysis

The cells were lysed in ice-cold RIPA buffer. Protein concentrations were determined using BCA Protein Assay Kit (Beyotime, China). Equal quantities of protein were separated using 10% SDS-polyacrylamide gels, transferred to a PVDF membrane and blocked with 5% bovine serum albumin (Amresco, USA). PVDF membranes were incubated overnight at 4 °C with the following antibodies: FN (Abcam, ab154210), CIP2A (Santa Cruz, sc-80662), β-catenin (CST, 9562), c-myc (CST, 5605), CyclinD1 (CST, 2922), and β-Actin (Santa Cruz, sc-47778). After washing, the membranes were then incubated with HRP-conjugated secondary antibodies followed by enhanced chemiluminescence detection.

### Construction of stably transfected cells

CIP2A short hairpin RNA (sh-CIP2A) plasmid and CIP2A overexpressed plasmid (CIP2A-plasmid) which were ligated in pGV101 vector and pGV142 vector respectively, were purchased from Shanghai GeneChem Technologies Co., Ltd. (Shanghai, China). The mature antisense sequence for shRNA we used is as following: 5’GATCCCCCACAGTTTAAGTGGTGGAAACTCGAGTTTCCACCACTTAAACTGTGGTTTTTGGAT-3’. These plasmids were transfected into T24 and J82 cells. Briefly, cells were inoculated into 6-well plates and incubated overnight. sh-CIP2A plasmid (4 μg) or CIP2A-plasmid (4 μg), was transfected in bladder cancer cells in 2 mL culture medium per well by using Lipofectamine 2000 (Invitrogen, USA) according to the manufacturer's protocol. In order to get stable cell lines, both CIP2A low expressed and CIP2A overexpressed cells were selected with 500 μg/mL neomycin for 14 days.

### RNA interference

The small interference RNAs (siRNAs) were purchased from GenePharma (Shanghai, China). The following sequences were used for RNA interference: β-catenin, 5’-CAG-GGGGUUGUGGUUAAGCUCUU-3’; negative control, 5’-UUCUCCGAACGUGU-CACGUTT-3’. Transfection of siRNAs (50 nmol/L) was conducted using Lipofectamine 2000 (Invitrogen, USA) according to the manufacturer's protocol.

### MTT assay

Cell viability was assessed using the 3-(4, 5-dimethylthiazol-2-yl)-2,5-diphenyltetrazolium bromide (MTT) assay, as previously described [19]. Cells were seeded on a 96-well plate and incubated with different concentrations of FN. At indicated time points after seeding, cell viability was determined by measuring the absorbance of MTT (Invitrogen, USA) at 490 nm using a microplate reader (MultiscanTMGO, Thermo, USA).

### Cell cycle analysis

The cell cycle analysis was performed by flow cytometry. Cells were collected and fixed with 70% ice ethanol overnight at 4 °C. The fixed cells were subsequently stained with propidium iodide (Beyotime, China). Cell cycle analysis was performed on a flow cytometer with the CellQuest software (BD Biosciences, USA).

### Immunohistochemical (IHC) analysis

The slides with paraffin-embedded tissues were deparaffinized in xylene and rehydrated through graded alcohols. Subsequently, 3% hydrogen peroxide was used to block the endogenous peroxidase activity for clear background staining. The slides were incubated with primary antibodies against FN (Abcam, ab154210), CIP2A (Santa Cruz, sc-80662), and PCNA (CST, 2586). The sections were washed 3 times with phosphate-buffered saline. Then, diaminobenzidine was used for signal development, and the slides were counterstained with 20% haematoxylin.

Estimation of scores for all samples was completed by the same pathologist in a blinded fashion which was performed according to the following semi-quantitative method. Slides with heavy background staining were excluded from the analysis. For the assessment of FN, each sample was scored based on the intensity of signal (negative staining = 0, weak staining = 1, moderate staining = 2, and strong staining = 3) and pattern (negative = 0, focal = 1, moderate = 2, diffuse = 3) of extracellular staining [20]. Then, we divided the specimens into two groups according to the sum of the two scores: low FN expression group (scores 0 to 3) and high FN expression group (scores 4 to 6). Moreover, the labelling index, which represented the percentage of immunostained cells relative to the total number of cells, was adopted for evaluating the levels of CIP2A and PCNA. Ten randomly chosen 400-fold high power fields and more than 500 cells were counted in each section were observed for analyzing.

### Immunofluorescence staining

Cells grown on coverslips in 24-well plates were fixed with 4% paraformaldehyde, permeabilized with 0.2% Triton X-100, and blocked with 5% bovine serum albumin (Amresco, USA). Then, the cells were incubated with antibodies against CIP2A (Santa Cruz, sc-80662) and β-catenin (CST, 9562) overnight at 4 °C, respectively. The secondary antibodies, anti-mouse Alexa Fluor 488 (Molecular Probes, A-11029) and anti-rabbit Alexa Fluor 594 (Molecular Probes, A-11037), were incubated with the cells at 37 °C for 1 h. The slips were washed 3 times, followed by staining with 4’,6-diamidino-2-phenylindole (DAPI) to visualize nucleus. Images were captured using a confocal laser-scanning microscope microscopy (Leica SP8, Germany).

### Co-immunoprcipitation (co-IP) assay

Cells were lysed by NP-40 lysis buffer with protease inhibitors. Meanwhile, Protein A Sepharose CL-4B beads (Sigma, Japan) were incubated with antibodies against β-catenin (CST, 9562) and normal rabbit IgG (designated as IgG, CST, 2729) at 4 °C for 4 h, respectively. Next, the beads were washed 3 times with NP-40 lysis buffer. Subsequently, the beads were mixed with cell lysates at 4 °C overnight. Then after washing beads for 6 times with NP-40 lysis buffer again, the bound proteins were eluted with SDS-PAGE loading buffer for western blotting analysis.

### Subcutaneous xenograft models

Animal experiments were performed according to the protocol approved by the Institutional Animal Care and Use Committee of Ruijin Hospital. Twenty 4-week-old female BALB/c nude mice were randomly divided into 4 equal groups. Sh-CIP2A and sh-Control T24 cells were incubated with FN (0 and 20 μg/mL) for 2 weeks, and then used for subcutaneous inoculations. Based on the various types of pretreatment, the four groups were named FN (-) sh-Control group, FN (-) sh-CIP2A group, FN (+) sh-Control group, and FN (+) sh-CIP2A group, respectively. The number of inoculated cells in each mouse was 2 × 10^6^. The mice in the two FN (+) groups were subcutaneously injected with 0.1 ml of FN solution (20 μg/mL) around the tumors every 2 days. Correspondingly, the mice were administered equal volume culture media in the two FN (-) groups. Tumor volume was measured using calipers every 7 days and calculated with the following formula: tumor volume (mm^3^) = (Length × Width^2^)/2. On day 35, the xenograft tumor tissues were removed and weighed. Afterwards, harvested tumor tissues were subjected to further IHC staining analysis.

### Statistical analysis

Data were expressed as the mean ± SD. Statistical analyses were conducted using SPSS 22.0 software (SPSS Inc., Chicago, USA). The correlation between FN and CIP2A expression in human bladder cancer tissues was analyzed using Spearman’s correlation test. The two-tailed Student *t* test was conducted to analyze statistical differences between groups. *P* values of less than 0.05 were considered statistically significant.

## Results

### FN expression correlates positively with the levels of CIP2A and PCNA in bladder cancer tissues

**Fig. 1:**
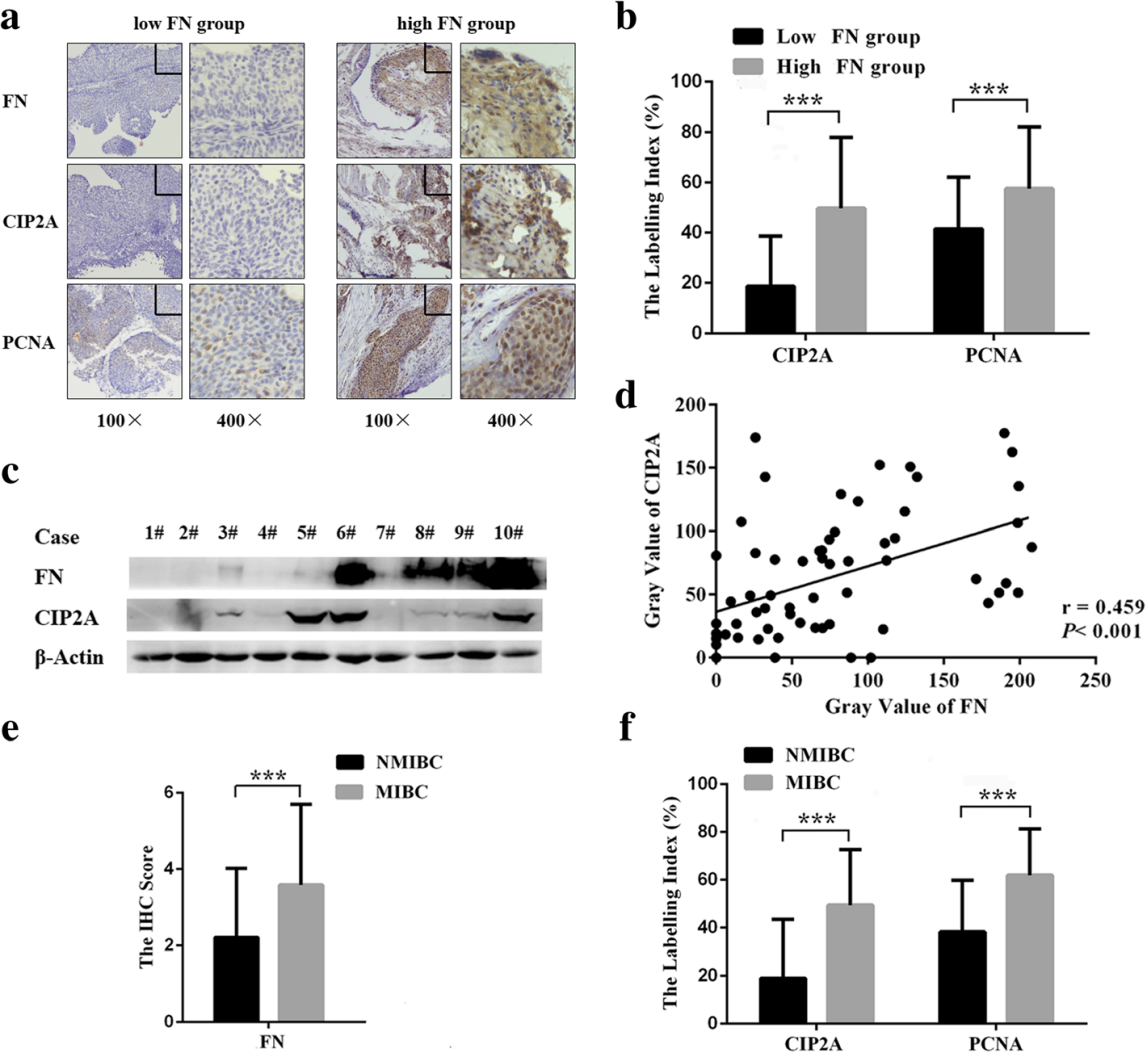
Stromal FN correlates positively with the levels of CIP2A and PCNA in bladder cancer tissues. **a** Representative photomicrographs of IHC staining for FN, CIP2A and PCNA in low FN group and high FN group were showed. **b** The labelling indexes of CIP2A and PCNA were assayed in low FN group and high FN group based on the IHC results (***P < 0.001). **c** Western blotting analysis of FN and CIP2A expression level was performed in bladder cancer tissues (*n* = 68). **d** *Gray value* of FN and CIP2A was measured by ImageJ software according to the western blotting results, and scatter plot with *regression line* showed a correlation of them using the Spearman’s correlation analysis. **e** and **f** According to the results of IHC, the expression of FN, CIP2A, and PCNA in NMIBC group and MIBC group were assayed (***P < 0.001)

The closely association between FN expression level and bladder cancer aggressivity has been validated [7]. Recent studies have shown that CIP2A enhances cell proliferative capacity in various malignant disorders, including bladder cancer [18, 21]. Therefore, we postulated that CIP2A mediated FN-induced bladder cancer cell proliferation. To examine the proliferative status of samples, sections of bladder cancer tissue were stained using antibody against PCNA which was widely used as a tumor marker for cancer cell proliferation [22]. Initially, we evaluated the association of the expression levels among FN, CIP2A and PCNA in bladder cancer tissue samples (*n* = 68). The IHC analysis illustrated that tissues derived from high FN group showed stronger staining of both CIP2A and PCNA than those in low FN group (Fig. 1a). Moreover, as shown in Fig. 1a, 400-fold high power fields showed that FN was predominantly localized in the peripheral stroma of malignant tumors and the remaining part was in cytoplasm of malignant urothelium, whereas positive staining of CIP2A and PCNA was mainly in cytoplasm and nucleus of cancer cells respectively. Based on the IHC results, the labelling indexes of CIP2A and PCNA in high FN group were both significantly higher than those in the low FN group (Fig. 1b). The following western blotting assays further strengthened the evidence linking FN with CIP2A in bladder cancer tissues (*n* = 68) (Fig. 1c), meanwhile, Spearman’s correlation analysis revealed the positive correlation between the gray value of CIP2A and PCNA (*r* = 0.459, *P* < 0.001) (Fig. 1d). Besides, the patients were categorized into either NMIBC group (*n* = 39) or MIBC group (*n* = 29) according to the pathological diagnosis. As expected, both the IHC score of FN (Fig. 1e) and the labelling indexes of CIP2A and PCNA (Fig. 1f) were significantly higher in MIBC cohort.

### FN induces cell proliferation and CIP2A expression in bladder cancer cells

**Fig. 2:**
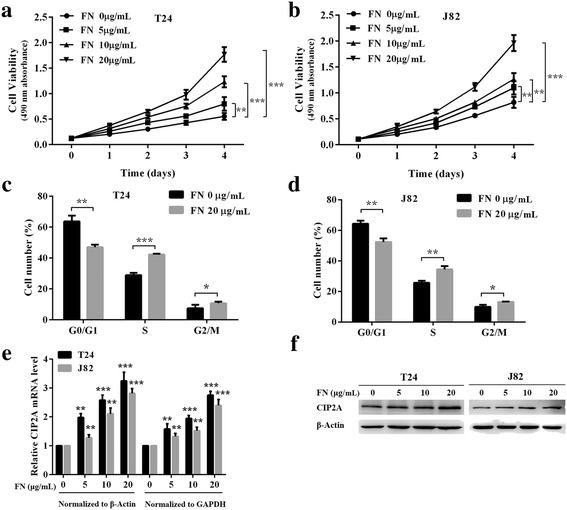
FN induces cell proliferation and CIP2A expression in bladder cancer cells. **a** and **b** Cell viability of T24 and J82 cells incubated with FN (0, 5, 10 and 20 μg/mL) for 4 days was evaluated by using MTT assay (**P < 0.01, ***P < 0.001). **c** and **d** Cell cycle distribution of T24 and J82 cells treated with FN (0 and 20 μg/mL) for 48 h was evaluated by flow cytometry. The percentage of cells in each phase were shown (*P < 0.05, **P < 0.01, ***P < 0.001). **e** and **f** CIP2A mRNA and protein expression levels of T24 and J82 cells were measured after FN treatment (0, 5, 10 and 20 μg/mL) for 48 h by qRT-PCR and western blotting respectively (**P < 0.01, ***P < 0.001)

To investigate the roles of exogenous FN in bladder cancer cell proliferation and CIP2A expression, MTT assay and flow cytometry assay were performed. MTT assay showed that FN obviously enhanced the proliferation ability of T24 and J82 cells in a dose-dependent manner (Fig. 2a and b). Furthermore, we estimated the effects of FN on cell cycle distribution by flow cytometry. Consistent with observed data in the MTT assay, after incubation with FN (FN 20 μg/mL), a decrease in the fraction of cells in the G0/G1 phase was observed in the both T24 and J82 cells, whereas the fraction of cells in the S and G2/M phases increased compared with the control group (FN 0 μg/mL) (Fig. 2c and d). Hence, these findings demonstrated that exogenous FN promotes bladder cancer cell proliferation.

We next focused on whether FN influenced CIP2A expression. Undergoing exogenous FN treatment, an increase of CIP2A levels emerged at both mRNA (Fig. 2e) and protein (Fig. 2f) levels, indicating that CIP2A expression was elevated by FN.

### CIP2A mediates FN-induced bladder cancer cell proliferation

**Fig. 3:**
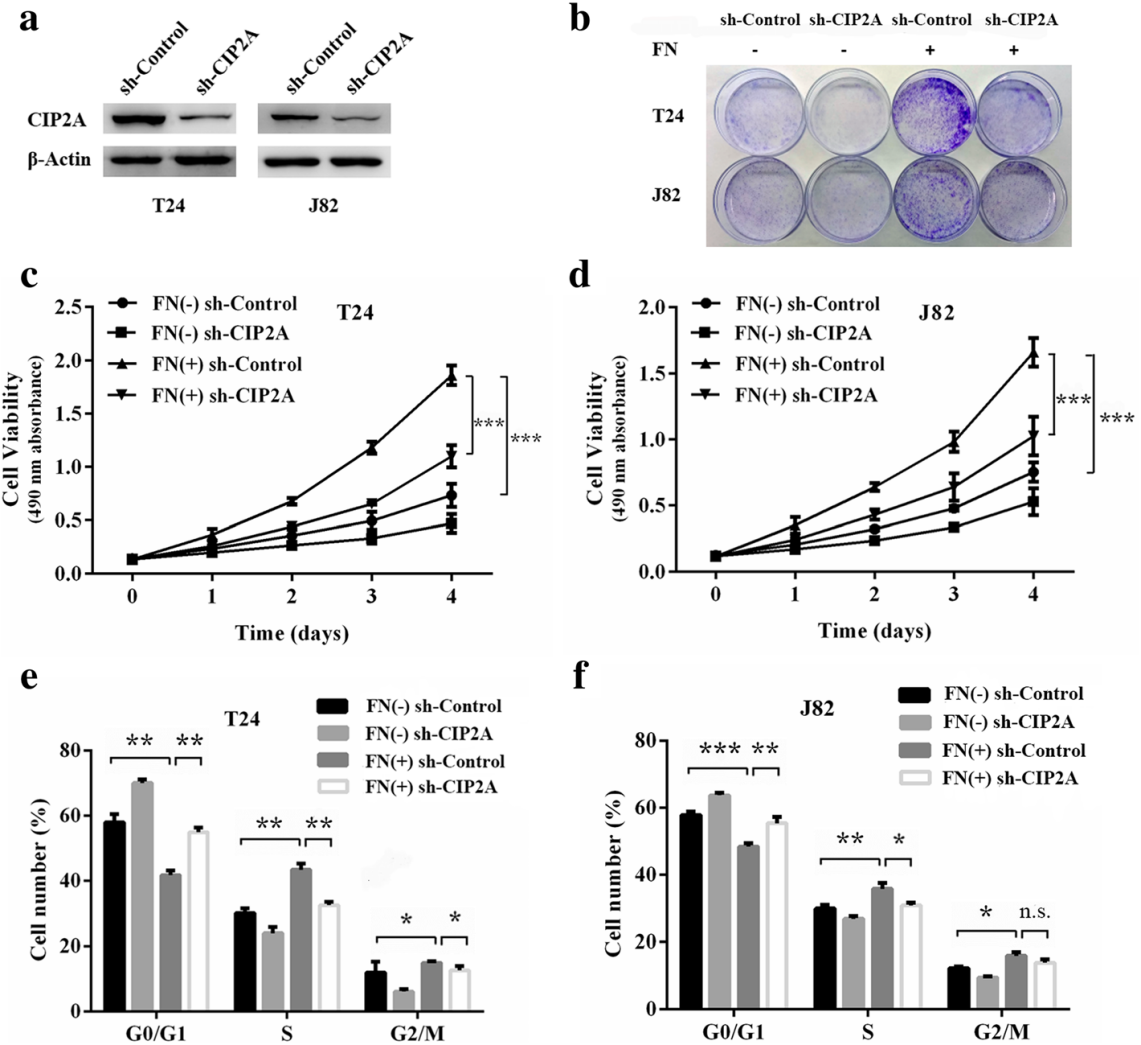
CIP2A mediates FN-induced bladder cancer cell proliferation. **a** CIP2A expression levels were examined in sh-CIP2A and sh-Control bladder cancer cells (T24 and J82). **b** sh-CIP2A and sh-Control bladder cancer cells (T24 and J82) were incubated with FN (0 and 20 μg/mL) for 2 weeks and allowed to form colonies. **c** and **d** The viability of sh-CIP2A and sh-Control bladder cancer cells (T24 and J82) incubated with FN (0 and 20 μg/mL) for 4 days was evaluated by using MTT assay (***P < 0.001). **e** and **f** Cell cycle distribution of sh-CIP2A and sh-Control bladder cancer cells (T24 and J82) treated with FN (0 and 20 μg/mL) for 48 h was evaluated by flow cytometry. The percentage of cells in each phase were shown (*P < 0.05, **P < 0.01, ***P < 0.001, n.s., not significant)

Based on the above findings, we assumed that FN may upregulate CIP2A to enhance cell proliferation. To define the functional links, MTT assay, colony formation and flow cytometry assay were performed by using sh-CIP2A and sh-Control bladder cancer cells. The western blotting results revealed that knockdown of CIP2A via sh-CIP2A in T24 and J82 cells substantially decreased the levels of CIP2A (Fig. 3a). CIP2A depletion in sh-CIP2A groups significantly abrogated FN induced elevation of the colony formation ability (Fig. 3b) and cell viability (Fig. 3c and d) in T24 and J82 cells. Moreover, compared with the FN-treated sh-Control cells, an increase in the fraction of cells in the G0/G1 phase was observed in the matched FN-treated sh-CIP2A T24 and J82 cells (Fig. 3e and f). Given that numerous proliferation-related signal pathways were activated by FN in other cancer models, we tested the effect of exogenous FN on CIP2A overexpressing bladder cancer cells to further ascertain that FN-mediated proliferation was primarily driven by increased CIP2A expression. The stably CIP2A-overexpressed T24 and J82 cells were validated by western blotting (Additional file 1: Fig. S1a). Subsequently, bladder cancer cells that overexpressed CIP2A proliferated fast, however, FN stimulation lacked the ability to enhance the proliferative capacity of CIP2A-overexpressed T24 and J82 cells significantly (Additional file 1: Fig. S1b and c). Taken together, these observations suggest that CIP2A mediates FN-induced bladder cancer cell proliferation. Taken together, these observations suggest that CIP2A mediates FN-induced bladder cancer cell proliferation.

### CIP2A is associated with β-catenin

**Fig. 4:**
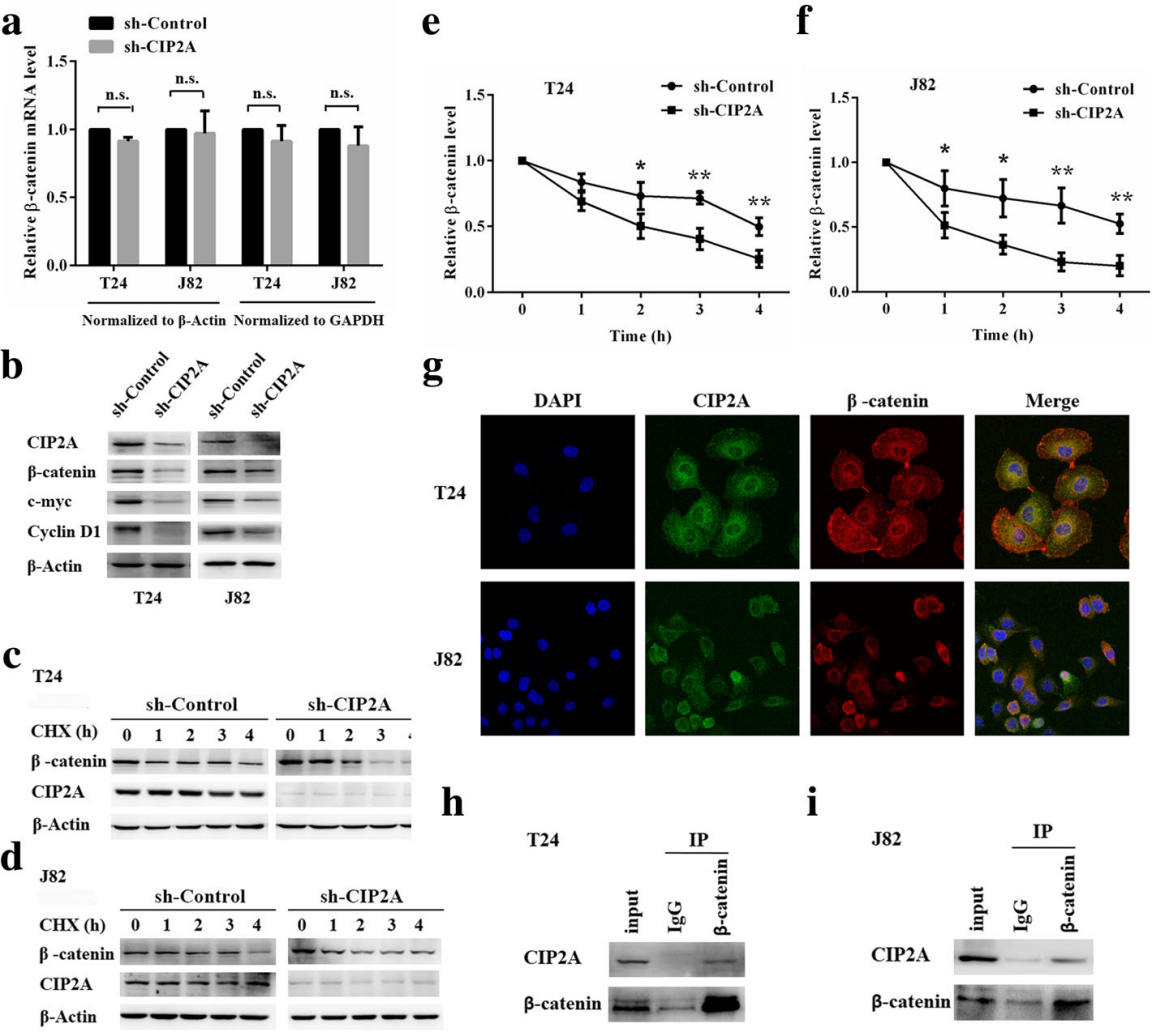
CIP2A is associated with β-catenin. **a** β-catenin mRNA levels were examined in sh-CIP2A and sh-Control bladder cancer cells (T24 and J82) (n.s., not significant). **b** CIP2A, β-catenin, c-myc, and CyclinD1 expression levels in sh-CIP2A and sh-Control bladder cancer cells (T24 and J82) were examined by western blotting. **c** and **d** sh-CIP2A and sh-Control bladder cancer cells (T24 and J82) were treated with CHX (100 μg/mL). At the indicated times (0, 1, 2, 3, and 4 h) after CHX treatment, cells were analyzed by western blotting. **e** and **f** The *gray value* of β-catenin were quantified using ImageJ software based on the western blotting results (*P < 0.05, **P < 0.01). **g** Immunofluorescence staining analysis showed the colocalization of CIP2A and β-catenin in T24 and J82 cells. **h** and **i** Co-IP analysis showed the interaction between CIP2A and β-catenin in T24 and J82 cells. CIP2A and β-catenin were immunoprecipitated using antibody against β-catenin. IgG was used as a negative control

Recent studies have confirmed that CIP2A is of paramount importance to maintain the activity of canonical Wnt/β-catenin pathway [23]. Hence, we hypothesized that CIP2A may regulate β-catenin. Based on the qRT-PCR results, there was no significant difference in β-catenin mRNA levels between sh-CIP2A cells and sh-Control cells (Fig. 4a). In contrast, expression of shRNA against CIP2A abolished the accumulation of both β-catenin protein and its targets, CyclinD1 and c-myc proteins (Fig. 4b), implying that CIP2A is involved in regulating β-catenin expression posttranscriptionally. Subsequently, we analyzed the half-life of β-catenin protein in sh-CIP2A cells and sh-Control cells subjected to CHX (100 μg/mL) incubation. Indeed, as shown in Fig. 4c and d, CHX treatment led to a sharper decrease of β-catenin in sh-CIP2A T24 and J82 cells. Consistently, quantification of β-catenin levels from three independent experiments revealed a statistically significant difference on β-catenin expression between CHX-treated sh-CIP2A cells and sh-Control cells (Fig. 4e and f). In sum, these results demonstrate that CIP2A increases the stability of β-catenin protein in bladder cancer cells.

Protein-protein interaction serves key regulatory roles to influence protein stability [17]. We, therefore, speculated that CIP2A may regulate β-catenin stability in such a manner. To test this, we detected the localization of the two proteins in T24 and J82 cells by immunofluorescence staining. As shown in Fig. 4 g, in general, CIP2A colocalized with β-catenin in both cytoplasm and nuclear. Furthermore, co-IP showed that CIP2A and β-catenin were seen in the cell lysate of T24 and J82 cells, and both proteins were immunoprecipitated with CIP2A antibody from cell lysate (Fig. 4 h and i), indicating that the CIP2A interacts with β-catenin in bladder cancer cells.

### β-catenin is involved in FN-induced bladder cancer cell proliferation

**Fig. 5:**
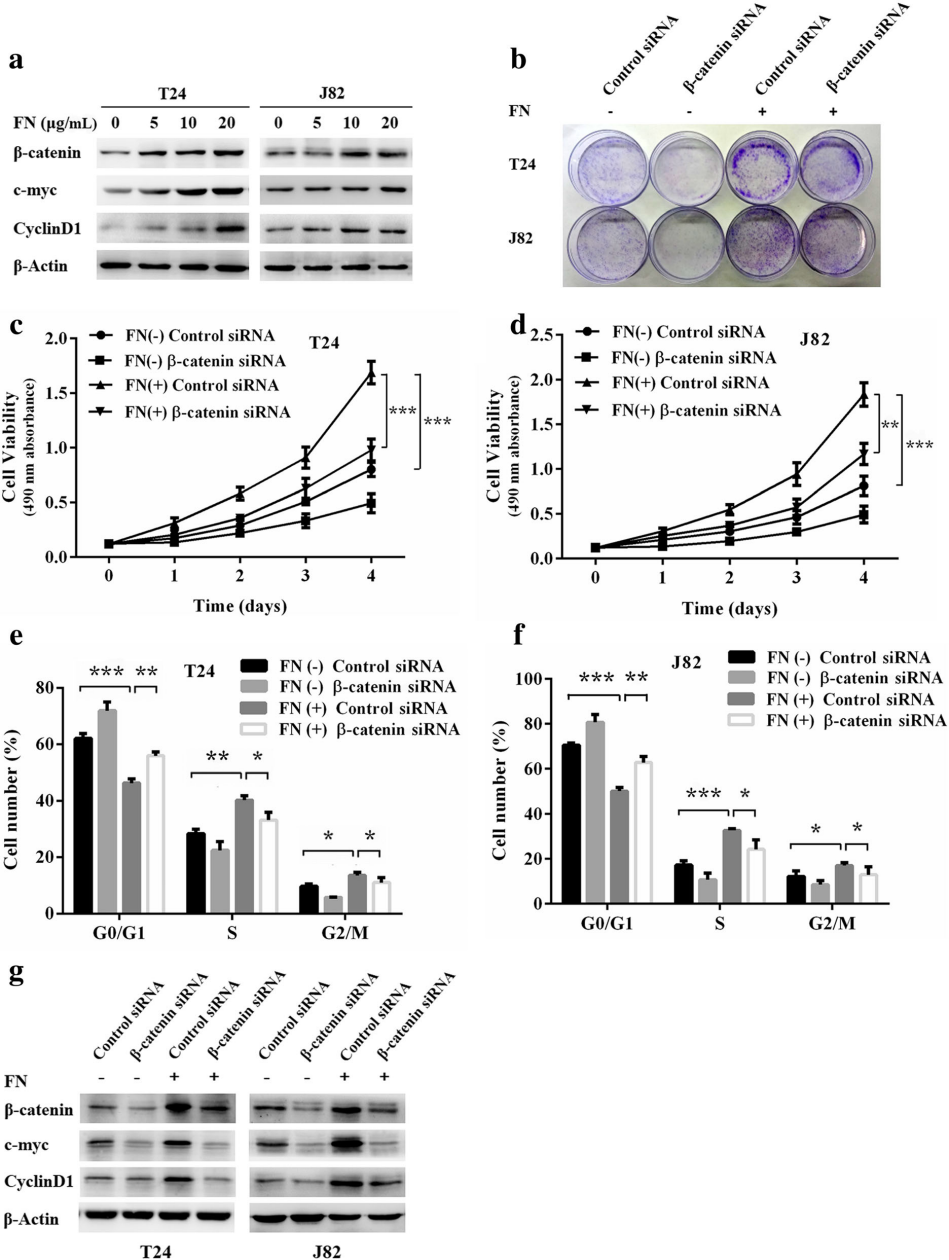
β-catenin is involved in FN-induced bladder cancer cell proliferation. **a** β-catenin, c-myc, and CyclinD1 expression levels in T24 and J82 cells were examined by western blotting after FN treatment (0, 5, 10 and 20 μg/mL) for 48 h. **b** T24 and J82 cells transfected with β-catenin siRNA or control siRNA were treated with FN (0 and 20 μg/mL) for 2 weeks and allowed to form colonies. **c** and **d** The viability of β-catenin siRNA or control siRNA transfected T24 and J82 cells which were incubated with FN (0 and 20 μg/mL) for 4 days was evaluated by using MTT assay (**P < 0.01, ***P < 0.001). **e** and **f** Cell cycle distribution of β-catenin siRNA or control siRNA transfected T24 and J82 cells treated with FN (0 and 20 μg/mL) for 48 h was evaluated by flow cytometry. The percentage of cells in each phase were shown (*P < 0.05, **P < 0.01, ***P < 0.001). **g** After 72 h transfection with β-catenin siRNA or control siRNA, T24 and J82 cells were treated with FN (0 and 20 μg/mL) for 48 h, and western blotting analysis of β-catenin, c-myc, and CyclinD1 expression levels were performed

Likewise CIP2A, β-catenin is also closely associated with cancer cell proliferation [24]. According to the above findings, we postulated the involvement of β-catenin in FN-induced bladder cancer cell proliferation. The western blotting assay results revealed that FN accelerated the accumulation of β-catenin, CyclinD1 and c-myc in a dose-dependent manner in both T24 and J82 cells (Fig. 5a). We next performed MTT assay and colony forming assay to determine whether β-catenin knockdown repressed cell proliferation caused by FN stimulation. Predictably, β-catenin depletion significantly weakened FN-induced the enhancement of colony formation ability (Fig. 5b) and cell viability (Fig. 5c and d) of bladder cancer cells. Furthermore, compared with control siRNA transfected cells, we observed a significant increase of the percentage of cells in the G0/G1 phase in β-catenin siRNA transfected cells (Fig. 5e and f). Consistent with the G1 phase arrest, the expression of CyclinD1 and c-myc decreased in cells transfected with β-catenin siRNA though the cells underwent FN stimulation (Fig. 5 g). Collectively, we confirmed that β-catenin participates in FN-induced bladder cancer cell proliferation.

### CIP2A mediates FN-induced bladder cancer cell proliferation in vivo

**Fig. 6:**
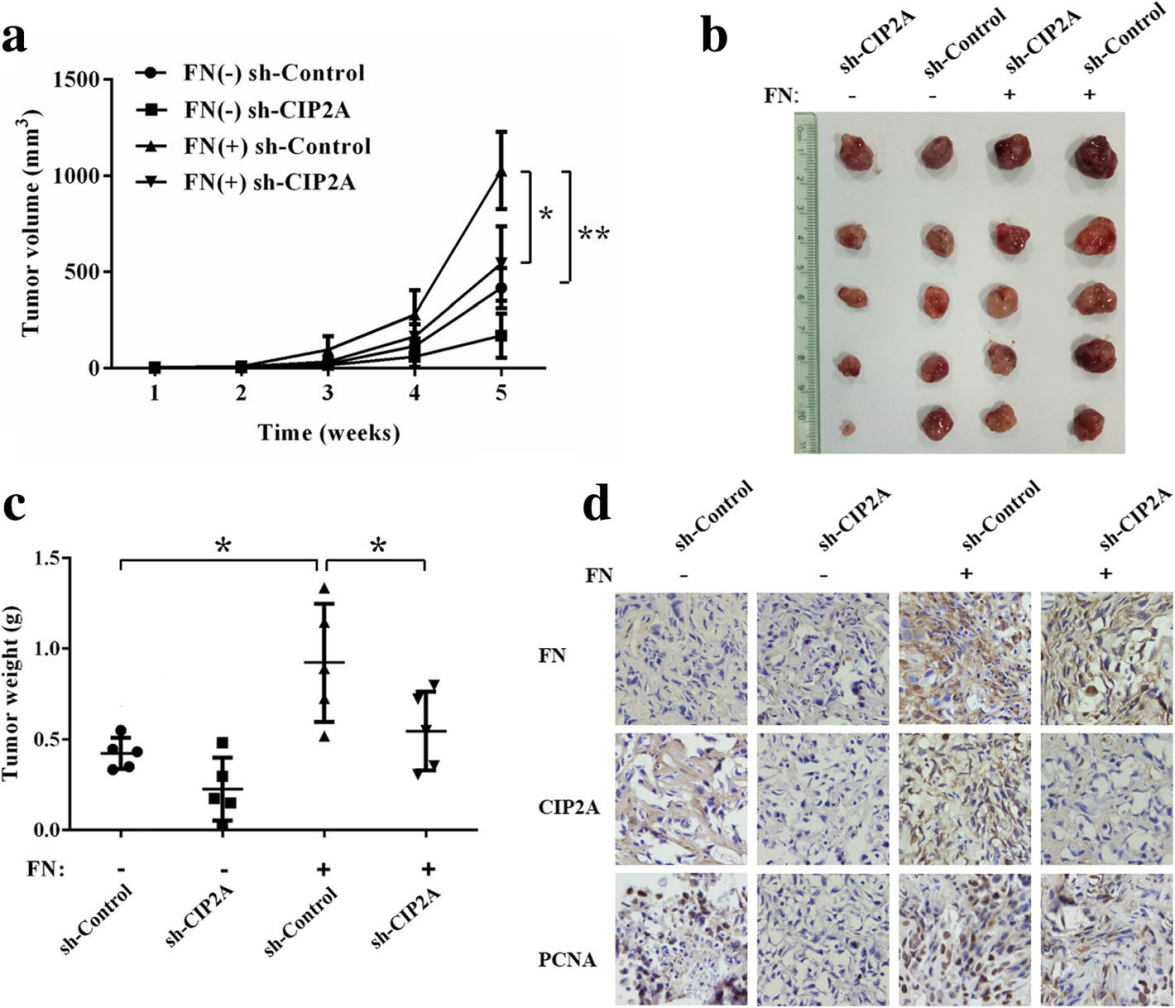
CIP2A mediates FN-induced bladder cancer cell proliferation in vivo. **a** Tumor growth curve of sh-CIP2A or sh-Control T24 cells’ bladder cancer subcutaneous xenograft tumors was built after FN or vehicle treatment for 5 weeks (*P < 0.05, **P < 0.01). **b** Photographs of dissected xenograft tumors from nude mice after sacrificed were presented. **c** The weight of dissected xenograft tumors in each group was assayed (*P < 0.05). **d** The IHC staining was performed to detect the expression levels of FN, CIP2A, and PCNA in harvested tumor tissues, and representative photomicrographs of 400-fold high power fields in each group were showed

To further confirm the proposed role of CIP2A in FN-induced cell proliferation of bladder cancer cells, we established subcutaneous xenograft models in BALB/c nude mice by using sh-CIP2A and sh-Control T24 cells. Neoplastic diameters were measured every week after inoculation to build the tumor growth curve. According to the tumor growth curve, in contrast with FN (+) sh-Control group, tumors in both FN (-) sh-Control group and FN (+) sh-CIP2A group grew at a slower rate (Fig. 6a), accompanied with significant reductions in tumor size (Fig. 6b) and weight (Fig. 6c). Moreover, IHC staining of the tumor tissues illustrated that percentage of PCNA positively stained cells in FN (+) sh-Control group was significantly higher than both FN (-) sh-Control group and FN (+) sh-CIP2A group (Fig. 6D). These results support that FN enhances the bladder cancer cells proliferative capacity, and CIP2A plays a critical role in the FN-induced cell proliferation in vivo.

## Discussion

Even though the overexpression and diagnostic value of FN in malignant bladder diseases have been previously reported, the biological function and its molecular mechanisms of FN in bladder cancer remain largely uncharacterized. In this study, we provided evidence on the link between stromal FN levels with cell proliferation in bladder cancer tissues. And both in vitro and in vivo experiments demonstrated that exogenous FN dramatically promoted cell proliferation and CIP2A expression in bladder cancer cells. Subsequently, we found that CIP2A interacted with β-catenin which contributing to β-catenin stabilization, and both the two proteins participated in FN-induced bladder cancer cell proliferation.

ECM presents distinct biochemical and biomechanical properties that facilitate cell growth, survival, adhesion and invasion in carcinogenesis [25]. As an important component of ECM, FN is a promising biomarker for diagnosis and treatment of bladder cancer. Studies have reported the overexpressed FN in high-grade bladder cancer [26], which coinciding with our IHC analysis results. In addition, FN is not only an integral feature of tumors but also actively forces cancer progression though complex signaling pathways. However, functional mechanisms of FN in bladder cancer cell biological behaviors have been little investigated. Consistent with the proliferating effects of FN in gallbladder cancer [27], our results revealed that FN remarkably promoted T24 and J82 cell proliferation. Hence, FN could appear to be a good candidate for bladder cancer treatment. Encouragingly, Huijbers et al. have developed a vaccine against FN to reduce tumor volume significantly [28]. Furthermore, the clinical evaluations of targeting FN have been ongoing. For example, Sauer et al. have reported that highly FN-specific radiolabeled antibody ^131^I-L19SIP induces a sustained partial remission in two Hodgkin lymphoma patients successfully, supporting the in vivo accessibility of this therapeutic approach in humans [29]. Besides, as regard to the source of stromal FN, previous observations suggest that FN could be secreted by tumor parenchymal cells [30]. And IHC results in our study identified the positive staining of FN in human bladder urothelial cells (Fig. 1A), indicating that bladder cancer parenchymal cells may be responsible for cancer-associated ECM remolding through producing and secreting FN during tumor progression. In accordance to the above evidences and assumptions, small molecules targeting intracellular FN synthesis and secretion could be effective to alter the components of tumor microenvironment.

The clinical relevance of CIP2A oncoprotein in bladder cancer aggressiveness has been established [31]. Similarly, our data demonstrated that MIBC tissues possessed higher levels of FN than NMIBC samples, and in addition, CIP2A promoted bladder cancer cell proliferation via abrogating G0/G1 arrest, which is similar to previous studies [21, 32]. Moreover, studies also confirm the expression of CIP2A varies with mitotic progression and is regulated by cell cycle-related factors [17, 33]. Besides, the influence of CIP2A on the expression of CyclinD1 and c-myc which is supported by our work also strengthens the close relationship between CIP2A and cell cycle.

Apart from promoting cell proliferation, the roles that FN and CIP2A played in bladder cancer deserve for further research. Due to the short postoperative time, lack of follow-up information hinders the exploration of the linkage between prognosis and FN or CIP2A expression for bladder cancer in our study. However, the prognostic prediction of FN and CIP2A have been confirmed in several other types of cancers [16, 34]. What’s more, studies also suggest that the two proteins are associated with anticancer drug resistance [10, 35]. Once better understandings of these mechanisms will be gained, FN and CIP2A may serve as potential biomarkers for poor survival and promising targets for increasing chemosensitivity.

β-catenin is responsible for regulating mitotic events [36]. Therefore, it is reasonable that β-catenin gets involved in FN-induced bladder cancer cell proliferation in our study. Meanwhile, we found that CIP2A stabilized β-catenin protein, though the exact mechanism is not yet clarified. Mechanistically, β-catenin can be phosphorylated by glycogensynthasekinase-3β (GSK-3β), but not phosphorylated GSK-3β (pGSK-3β) [36]. Then the phosphorylated β-catenin (p-β-catenin) is presented to the proteasome for degradation [37]. Consequently, interfering with the formation of p-β-catenin is an important approach to elevate β-catenin stabilization. We found that CIP2A stabilized β-catenin protein, though the exact mechanism was not yet clarified. With further research, we explored and validated the colocalization and interaction between CIP2A and β-catenin, indicating that the CIP2A-β-catenin interaction may repress the process of β-catenin phosphorylation. Protein phosphatase 2A (PP2A), whose catalytic phosphatase activity can be inhibited by CIP2A, has been reported to repress β-catenin abundance [38]. Consistently, studies show that PP2A induced dephosphorylation of pGSK-3β, thereby promoting β-catenin degradation [23]. Based on the above evidence, CIP2A may contribute to β-catenin accumulation by suppressing the phosphatase functions of PP2A to modulate GSK-3β activation in bladder cancer cells.

## Conclusions

In this study, we provide evidence that CIP2A is involved in FN-induced bladder cancer proliferation by enhancing β-catenin stabilization. Moreover, we find CIP2A interacts with β-catenin which may be responsible for β-catenin stabilization. Therefore, FN-CIP2A-β-catenin signaling pathway may be a novel suitable target for clinical intervention in bladder cancer patients.

## Additional file

Additional file 1: Figure S1. Exogenous FN has no significant effect on CIP2A-overexpressed bladder cancer cells proliferation. a CIP2A expression levels were examined in CIP2A-plasmid and Control-plasmid bladder cancer cells (T24 and J82). b and c The viability of CIP2A-plasmid and Control-plasmid bladder cancer cells (T24 and J82) incubated with FN (0 and 20 μg/mL) for 4 days was evaluated by using MTT assay (**P* < 0.05, ***P* < 0.01, ****P* < 0.001, n.s., not significant). (TIF 840 kb)

## Abbreviations

CHX: Cycloheximide
CIP2A: Cancerous inhibitor of protein phosphatase 2A
co-IP: co-immunoprcipitation
ECM: Extracellular matrix
FN: Fibronectin
IHC: Immunohistochemistry
MIBC: Muscle-invasive bladder cancer
MTT: 3-(4,5-dimethylthiazol-2-yl)-2,5-diphenyltetrazolium bromide
NMIBC: Non-muscle-invasive bladder cancer
PCNA: Proliferating cell nuclear antigen
PP2A: Protein phosphatase 2A
qRT-PCR: quantitative real-time PCR
DAPI: 4’,6-diamidino-2-phenylindole
sh-CIP2A: Short hairpin RNA
siRNA: Small interference RNA
TURBT: Transurethral resection of bladder tumor
