# Supplementary figures and images for: CIP2A mediates fibronectin-induced bladder cancer cell proliferation by stabilizing β-catenin
Source: J Exp Clin Cancer Res. 2017 May 18;36:70. doi: 10.1186/s13046-017-0539-8 (PMC5437599; doi:10.1186/s13046-017-0539-8)

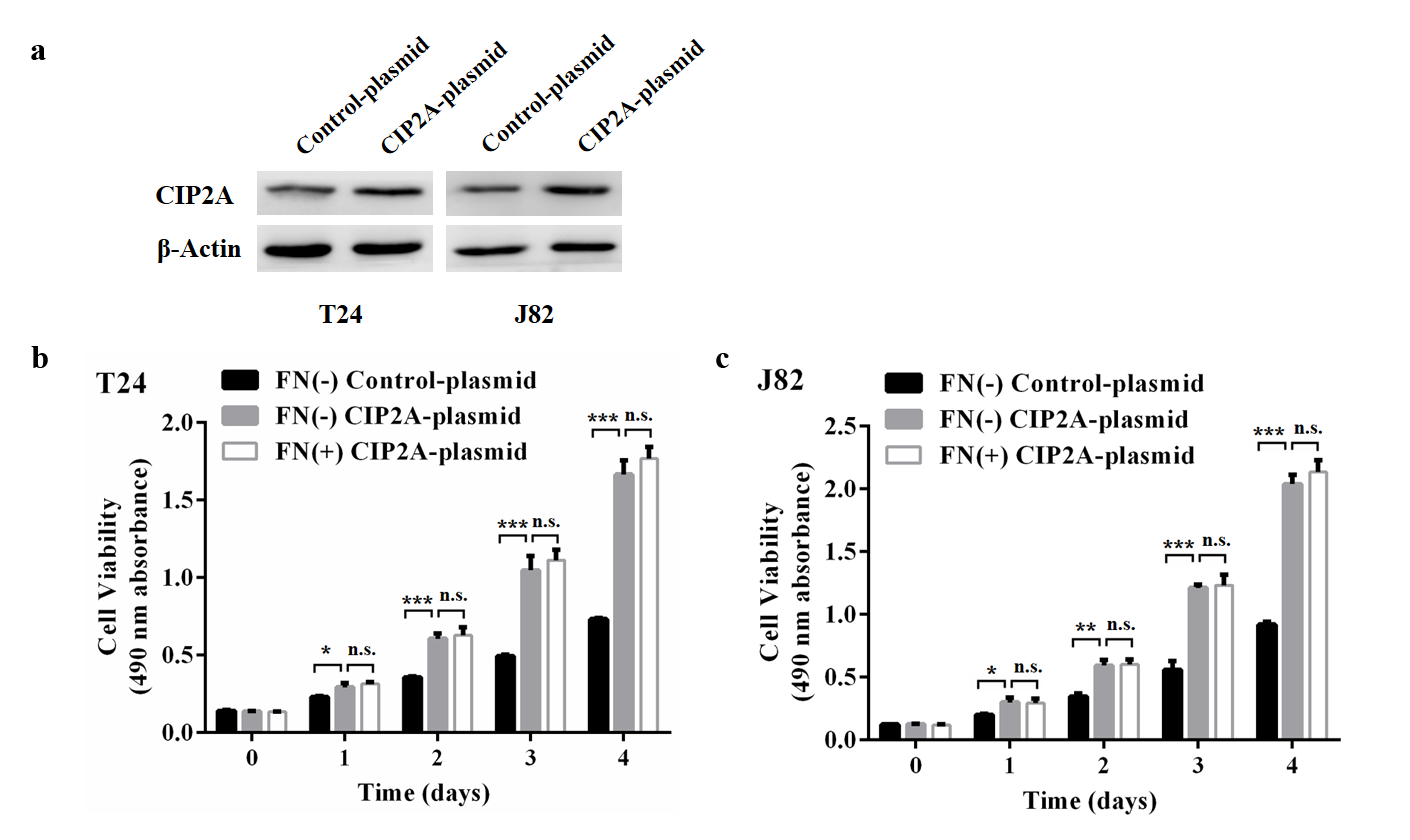

Supplement: Additional file 1: Figure S1. — Exogenous FN has no significant effect on CIP2A-overexpressed bladder cancer cells proliferation. a CIP2A expression levels were examined in CIP2A-plasmid and Control-plasmid bladder cancer cells (T24 and J82). b and c The viability of CIP2A-plasmid and Control-plasmid bladder cancer cells (T24 and J82) incubated with FN (0 and 20 μg/mL) for 4 days was evaluated by using MTT assay (*P < 0.05, **P < 0.01, ***P < 0.001, n.s., not significant). (TIF 840 kb) [file 13046_2017_539_MOESM1_ESM.tif]
